# In Vivo Effects of Neonicotinoid-Sulfoximine Insecticide Sulfoxaflor on Acetylcholinesterase Activity in the Tissues of Zebrafish (*Danio rerio*)

**DOI:** 10.3390/toxics9040073

**Published:** 2021-04-01

**Authors:** Petek Piner Benli, Mehmet Çelik

**Affiliations:** 1 Department of Veterinary Pharmacology and Toxicology, Faculty of Ceyhan Veterinary Medicine, Cukurova University, Adana 01330, Turkey; 2 Department of Veterinary Food Hygiene and Technology, Faculty of Ceyhan Veterinary Medicine, Cukurova University, Adana 01330, Turkey; mcelik@cu.edu.tr

**Keywords:** neonicotinoids, sulfoxaflor, LC_50_, AChE, brain, muscle, zebrafish

## Abstract

Sulfoxaflor is the first member of the neonicotinoid-sulfoximine insecticides that acts as an agonist of nicotinic acetylcholine receptors (nAChRs). This study investigated the acute effects of sulfoxaflor on acetylcholinesterase (AChE; EC 3.1.1.7) enzyme activity in the brain and muscle tissues of zebrafish (*Danio rerio*) as a model organism. The zebrafish were exposed to 0.87 mg/L (2.5% of 96 h 50% lethal concentration (LC_50_), 1.75 mg/L (5% of 96 h LC_50_) and 3.51 mg/L (10% of 96 h LC_50_) of sulfoxaflor for 24 h–48 h and 96 h periods. AChE enzyme activities were analysed by a spectrophotometric method in the brain and muscle tissues. The results of this study showed that in vivo acute sulfoxaflor exposure significantly increased AChE enzyme activity in the brain and muscle tissues of zebrafish. The induction percentages of AChE were between 10 and 83%, and 19 and 79% for brain and muscle tissues, respectively. As a result, it was found that sulfoxaflor had an effect on AChE enzyme activity in the two main tissues containing this enzyme, and it can be considered as a potential neuroactive compound for zebrafish.

## 1. Introduction

Sufoxaflor[methyl(oxo){1-[6-(trifluoromethyl)-3-pyridyl]ethyl}-λ6-sulfanylidene]cyanamide (IUPAC) (Chemical Abstracts Service No. 946578-00-3) is one of the newly developed neonicotinoid-sulfoximine insecticides [1] and it acts as a nicotinic acetylcholine receptor (nAChR) agonist in insects [2]. Sulfoxaflor has a unique structure–activity relation when compared with other neonicotinoids, since it contains the sulfoximine group. Sulfoximines are effective in the nAChRs of insects, like other neonicotinoids, but differ from other neonicotinoids while interacting with other nAChRs [1]. Recent studies reported that sulfoxaflor is highly toxic to some aquatic organisms [3] and bees [4,5,6]. Furthermore, sulfoxaflor also has carcinogenic [7,8] and teratogenic effects on mammals [9].

The use of neonicotinoids has increased in the global market during the last two decades [10,11,12]. Studies have reported that neonicotinoids have certain adverse effects on wildlife, considering direct (toxic) or indirect (e.g., food chain) impacts on birds, amphibians, fish, reptiles and mammals [13]. Neonicotinoids interfere in neural transmission in the central nervous system and hence they cause neurotoxicity. While neonicotinoids are highly selective on insect nicotinic receptors, a number of studies have shown that the compounds can activate and/or modulate the nicotinic receptors of humans [14] and other vertebrates [15,16,17,18]. The metabolites of some neonicotinoids can have higher affinity with mammalian nAChRs, similar to nicotine [19]. It has been known that neonicotinoids poorly penetrate the blood–brain barrier (BBB) [20]. In contrast, recent studies have demonstrated that specific neonicotinoids or their metabolites may lead to neurotoxic effects in model mammals [21,22,23,24]. Furthermore, neonicotinoids including thiacloprid, acetamiprid, nitenpyram and imidacloprid could freely pass through the BBB and could be detectable in the brain of mice [25]. It is also possible that some neonicotinoids such as acetamiprid could pass through the BBB and accumulate in the brain [26]. However, there is no evidence related to the penetration of sulfoxaflor through the BBB.

The neurotoxicity potential of pesticides can be determined by alterations in the cholinesterase (ChE) activities in the different tissues of organisms [27,28]. ChEs are divided into two main groups: acetylcholinesterase (AChE; EC 3.1.1.7) and butyrylcholinesterase (BChE; EC 3.1.1.8). The primary physiological function of AChE is the breakdown of acetylcholine (ACh), which mediates cholinergic synapses during the transmission of nerve impulses [29,30]. Recent research has showed that some neonicotinoids can inhibit AChE enzyme activity in fish [31,32] and mammals [22,24,33], but others may cause an induction in AChE enzyme activity in bees [34,35], arthropods [36] and fish [37]. There is no report related to the effects of sulfoxaflor on AChE activity in the fish tissues. 

Zebrafish (*Danio rerio*) express AChE in the brain and muscle tissues, and are selected as a model organism in this research, with no detectable butyrylcholinesterase (BChE) activity [38]. The human and zebrafish AChE enzymes also have almost 62% similar amino acid sequences [39]. The zebrafish has been proposed as a well-established model organism in toxicological research, with a number of studies evaluating its role as an important nonmammalian model for neurotoxicity of xenobiotics [40,41,42,43]. This study was carried out to determine the acute effects of sulfoxaflor on AChE enzyme activity in the brain and muscle tissues of zebrafish, as a model organism.

## 2. Materials and Methods

### 2.1. Chemicals

A commercially available sulfoxaflor (Chemical Abstracts Service, CAS number: 946578-00-3, [methyl(oxo){1-[6-(trifluoromethyl)-3-pyridyl]ethyl}-λ6-sulfanylidene]cyanamide) formulation called Transform 500 WG (50% *w*/*w* sulfoxaflor active ingredient, 20–30% *w*/*w* porcelain clay, 10–20% *w*/*w* urea polymer with formaldehyde, <5% *w*/*w* sodium N-methyl-N-oleoyltaurine) [44] was obtained from a distributor company in Turkey (Dow AgroSciences, Istanbul, Turkey). All chemicals (analytical grade, 95–98% purity) were purchased from Sigma-Aldrich Co. (St. Louis, MO, USA) and Merck & Co. Inc. (Merck, Darmstadt, Germany) for measuring AChE enzyme activity and protein levels in tissues.

### 2.2. Animals and Test Conditions

Adult mixed sex zebrafish (*D. rerio*) of the wild type (half female, half male) (0.58 ± 0.12 g weight) were commercially supplied. Fish were maintained for 2 weeks in a renewal static system in 100 L glass aquaria with a 14 h/10 h light/dark cycle at 28 °C ± 1 °C during the adaptation periods. Aquaria water was continuously aerated using a static pump system. The physiochemical properties of aquaria water (dissolved oxygen, 6.87 ± 0.75 mg/L; pH, 7.63 ± 0.5; temperature, 28.23 ± 0.82 °C; alkalinity, 245 ± 3.59 mg/L as CaCO_3_; total hardness, 252 ± 11.55 mg/L as CaCO_3_) were recorded regularly. Stock fish were fed with commercial fish pellets twice a day during the adaptation periods. Feeding was stopped 24 h before the toxicity test. Experimental procedures were conducted in accordance with the protocols approved by the Ethics Committee of the Çukurova University Faculty of Medicine Experimental Medicine Research and Application Centre (approval code: 3; approval date: 4 July 2018). All toxicity tests were performed with a renewal static system in accordance with the American Public Health Association’s guidelines [45]. The stock solution of sulfoxaflor used in this study was freshly prepared from distilled water. Water in the aquaria was changed at 24 h intervals by transferring the fish to other aquaria.

### 2.3. Determination of the 50% Lethal Concentration Value of Sulfoxaflor in *Zebrafish*

Randomly selected fish were divided into 6 groups (1 control group and 5 treatment groups). There were 12 fish in each group. Each group was transferred into 20 L individual glass aquaria in order to determine the 96 h 50% lethal concentration (LC_50_) value of sulfoxaflor. The whole experiment was replicated 2 times so that each group was assigned 24 fish. Preliminary tests were conducted to determine the average dose range. One aquarium was set as a control, and 5 different nominal sulfoxaflor concentrations (24.1, 28.13, 32.14, 40.50 and 49.94 mg/L) were applied to the other aquaria. The experiments were conducted for 96 h. Dead fish were recorded per group during the test procedures. At the end of the 96 h exposure period, the LC_50_ value of sulfoxaflor was determined as 35.13 mg/L (95% confidence interval (CI), 32.469–38.298) by using probit analysis.

### 2.4. Acute Toxicity Tests

Acute toxicity tests were carried out in 4 separate 60 L glass aquaria. The selected fish were randomly divided into 4 different experimental groups (Group I: control; Groups II, III and IV: treatment). Each experimental group comprised 108 individuals: 36 fish for the 24 h exposure period, 36 fish for the 48 h exposure period and 36 fish for the 96 h exposure period. Furthermore, each experimental unit consisted of a pool of 6 individuals, from which the required amount of tissue samples was collected and pooled for each individual measurement. Thus, the measurements were repeated in 6 technical replicates (*N* = 6). Sulfoxaflor concentrations were selected considering toxicity symptoms such as loss of balance, erratic swimming and rapid gill movement based on preliminary tests. In preliminary tests, fish were exposed to 5 different concentrations of sulfoxaflor for 96 h and toxicity symptoms were observed after exposure to >3.51 mg/L (10% of 96 h LC_50_) sulfoxaflor without mortality. Three sublethal concentrations were chosen, 0.87 mg/L (2.5% of 96 h LC_50_), 1.75 mg/L (5% of 96 h LC_50_), 3.51 mg/L (10% of 96 h LC_50_), for the acute toxicity test. Group I was held in clean water as a control. Groups II, III and IV were exposed to 0.87 mg/L (2.5% of 96 h LC_50_), 1.75 mg/L (5% of 96 h LC_50_) and 3.51 mg/L (10% of 96 h LC_50_) of sulfoxaflor for 24 h–48 h and 96 h. Fish were removed from the aquaria at the end of each test period. Fish were weighed and quickly euthanized by decapitation. The brain and muscle tissues of the fish were carefully dissected out on an ice plate before washing them with saline, weighing and storing them at −80 °C until the analysis was completed.

### 2.5. Preparation of Tissue Homogenates

A pool of 6 brain and muscle tissues from fish was homogenized separately in an ice-cold 0.1 M phosphate buffer (pH 7.4, containing Triton-X 100). The homogenates were centrifuged (Hettich Micro 220, Tuttlingen, Germany) at 13,000× *g* for 30 min at +4 °C and the supernatants were used to determine AChE enzyme activities and protein levels.

### 2.6. Determination of Acetylcholinesterase Enzyme Activity

AChE activities in brain and muscle tissues were detected by using the spectrophotometric method (Shimadzu UV-Vis Spectrophotometer UV-1700, Kyoto, Japan) according to the modified method [31] developed by Ellman [46]. Increases in absorbance at 412 nm were measured for 4 min at 25 °C in the presence of 0.5 mol/L phosphate buffer (pH 8.0), 10 mmol/L acetylcholine iodide, 0.5 mmol/L 5,5-dithio-2-dinitrobezoic acid (DTNB) (in 1% sodium citrate). Specific enzyme activities were calculated as U/mg protein using a substrate free blank [46].

### 2.7. Determination of Protein Levels

The protein levels of homogenates obtained from brain and muscle tissues were determined by utilizing the method developed by Bradford [47]. For this, 100 µL of the diluted homogenates was added to 3 mL of Bradford reagent, then the mixtures were incubated for 30 min at room temperature. The absorbances were recorded at 595 nm using a UV-Vis spectrophotometer (Shimadzu UV-Vis Spectrophotometer UV-1700). Protein amounts were calculated from the standard graph prepared using bovine serum albumin.

### 2.8. Statistical Analysis

The LC_50_ value was determined by probit analysis using the SPSS 22.0 package program. All data are denoted as means ± standard error. Analysis of variance (one-way ANOVA) and Duncan’s multiple comparison tests were used to determine statistical differences between the control and test groups using the SPSS 22.0 package program.

## 3. Results

### 3.1. Determination of the 96 h LC_50_ Value of Sulfoxaflor for Zebrafish

The acute toxicity test showed no mortality in control fish. In the sulfoxaflor-exposed groups, the recorded mortalities were 24.1, 28.13, 32.14, 40.50 and 49.94 mg/L concentrations, while the percentage of mortality was 4%, 29%, 45%, 54% and 87%, respectively, after 96 h of exposure. The 96 h LC_50_ of sulfoxaflor was calculated as 35.13 mg/L for zebrafish following probit analysis (95% confidence interval (CI), 32.469–38.298, *p <* 0.05) (Table 1).

### 3.2. Changes in AChE Enzyme Activity in the Brain

**Figure 1:**
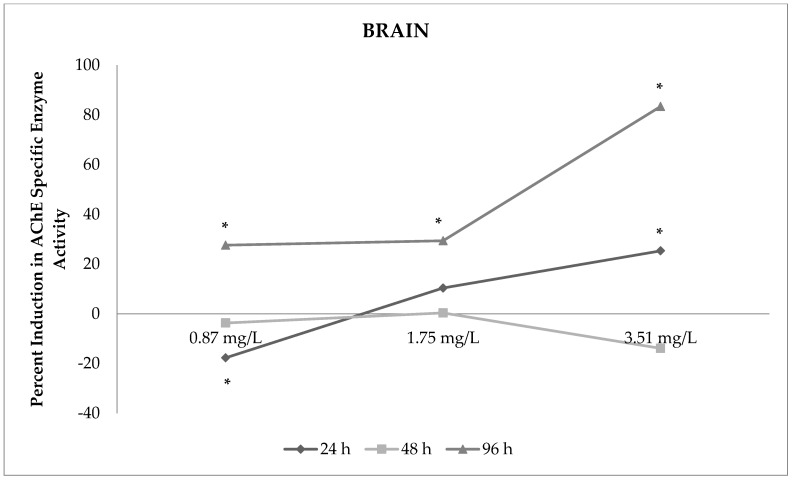
Percent induction in AChE-specific enzyme activity in the brain of sulfoxaflor-exposed zebrafish. * Percent induction in AChE-specific enzyme activity is significant compared with the control.

Sulfoxaflor significantly increased the activity of AChE enzymes in the brain of zebrafish (from 10% to 83%; *p* < 0.05). The results indicated that the 96 h exposure period had a greater effect on AChE enzyme activity compared with other exposure periods in the brain. AChE enzyme activities rose significantly with higher concentrations of sulfoxaflor in the brain (*p* < 0.05). The activity of AChE enzymes decreased by 17% with 0.87 mg/L sulfoxaflor exposure and increased by 10% and 25% with 1.75 mg/L and 3.51 mg/L sulfoxaflor exposure at 24 h, respectively (*p* < 0.05). In addition, inductions in AChE activity were determined at 96 h for all tested sulfoxaflor concentrations. AChE enzyme activity was induced by 27%, 29% and 83% with 0.87 mg/L, 1.75 mg/L and 3.51 mg/L, sulfoxaflor exposure at 96 h, respectively (Table 2, Figure 1; *p* < 0.05).

### 3.3. Changes in AChE Enzyme Activity in Muscles

**Figure 2:**
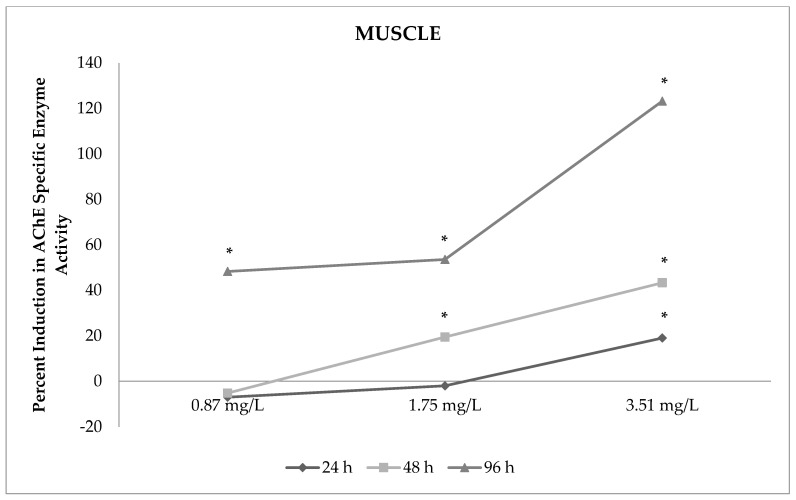
Percent induction in AChE-specific enzyme activity in the muscles of sulfoxaflor-exposed zebrafish. * Percent induction in AChE-specific enzyme activity is significant compared with the control.

AChE enzyme activity was significantly increased by sulfoxaflor in the muscles of zebrafish (from 19% to 79%; *p* < 0.05). The results indicated that AChE enzyme activity was significantly increased by high concentrations of sulfoxaflor during all of the exposure periods in the muscle (Table 3, Figure 2; *p* < 0.05). Sulfoxaflor exposure significantly increased AChE enzyme activity by 19% for 3.51 mg/L concentration at 24 h (*p* < 0.05). Similarly, AChE enzyme activity was induced by 21% and 24% with 1.75 mg/L and 3.51 mg/L sulfoxaflor exposure at 48 h, respectively (*p* < 0.05). The activity of AChE enzymes was increased by 53%, 34%, and 79% by 0.87 mg/L, 1.75 mg/L and 3.51 mg/L sulfoxaflor exposure at 96 h, respectively (*p* < 0.05). The results demonstrated that the elevations in AChE enzyme activity were not dependent on the sulfoxaflor concentrations and exposure periods in the muscle (Table 3, Figure 2). Increasing exposure time caused significantly higher AChE enzyme activity at 1.75 mg/L and 3.51 mg/L sulfoxaflor concentrations in the muscle. In addition, the 96 h exposure period had a greater effect on AChE enzyme activity compared with other exposure periods in the muscle.

## 4. Discussion

Based on previous research into the effects of neonicotinoids on other nontarget organisms, the present study investigated the effects of acute exposure to sulfoxaflor on zebrafish brain and muscle by evaluating the AChE activity. The AChE activity of brain and muscle, one of the biomarkers of toxicity, is a beneficial measure to examine the possible action of toxicants [27,28,31,32,37]. In this study, significant induction was determined by sulfoxaflor exposure; however, AChE induction might not be suggested as a biomarker due to the lack of a dose–response relationship for sulfoxaflor exposure. In fish, AChE is predominant in brain and muscle tissues [48]. Recent studies showed that toxicants have a greater effect on AChE in brain and muscle tissues than other tissues of zebrafish [49,50]. The tissue-specific response was not determined due to similar induction rates of AChE by sulfoxaflor in both tissues of zebrafish.

AChE can be considered as a pivotal enzyme which breaks down the neurotransmitter acetylcholine into choline and acetate, and therefore pesticides target this enzyme [33,51]. The present study showed that sulfoxaflor caused elevations in AChE enzyme activity in the brain and muscle tissues of zebrafish with acute exposure. Recent studies showed that neonicotinoids caused an inhibition of AChE enzyme activity in the different tissues of fish species [31,32] and mammals [22,24,33]. Previously, we determined that spinosad, which acts as an agonist of nAChRs, inhibited AChE enzyme activity in the liver and brain tissues of *Oreochromis niloticus* [52]. The mechanism of modulation of ChE by neonicotinoids remains to be completely clarified. However, certain possible mechanisms that inhibit AChE are addressed by in vitro research [53,54]. The inhibitory effects of thiamethoxam, clothianidin, acetamiprid and thiacloprid on purified eel AChE were identified, where the concentration was dependent on other dynamics and the four tested neonicotinoids varied in their blocking ability. The authors suggested that the neuronal AChE enzyme is likely to be among the direct targets of the neonicotinoid insecticides [53]. Terali et al. [54] demonstrated that the seven neonicotinoid insecticides (namely acetamiprid, clothianidin, dinotefuran, imidacloprid, nitenpyram, thiacloprid and thiamethoxam) have the potential to inhibit human ChEs using in silico analyses; however, it is predicted that these might choose and include various binding modes in the active-site gorge of AChE. In contrast to these findings, AChE enzyme activity was induced by the neonicotinoid insecticide imidacloprid in the brain of *Gobiocypris rarus* [37]. Consistent with this research, it was found that acute sulfoxaflor exposure caused increases in AChE enzyme activity between 10–83% and 19–79% in the brain and muscle tissues of zebrafish in the present study. It was suggested sulfoxaflor could have a relative impact on the nAChRs in brain and muscle tissues of zebrafish, and hence the AChE activity could be induced to diminish excess acetylcholine at the cholinergic synaptic clefts. The AChE induction mechanism of sulfoxaflor and its metabolites has not been determined yet in vitro and in vivo. Zhang et al. [55] demonstrated that pharmacological inhibitors of AChE prevented apoptosis and suggested that induction of AChE is a possible marker and regulator of apoptosis. Jin et al. [56] reported that increases in AChE activity are likely to hinder cell proliferation and elevate apoptosis in the brain tissues.

The tests related to the effects of pesticides on behavioural alterations in animals are necessary for evaluating their neurotoxic effects and their effects on the endocrine system [57]. These behavioural alterations consist of changes in locomotor activity, eating behaviour, attack or avoidance behaviour and reproductive behaviour in fish [58]. Other studies reported that alterations in AChE enzyme activity induced by pesticides caused changes in fish behaviour [59,60,61,62,63]. Neonicotinoids cause a modulation in AChE enzyme activity, mostly by inhibiting this specific molecular target of various pesticides in fish [31,32] and mammals [22,24,33]; hence neonicotinoids are most likely to alter the behaviour of mammals [21,22,24]. Gestational administration of imidacloprid caused significant increases in AChE activity in different brain regions of rats, and it produced neurobehavioural changes depending on sensorimotor impairments that were reflected in the beam walk time, inclined plane performance and forepaw grip in male and female offspring [21]. Systemic administration of thiamethoxam resulted in inhibition of AChE enzyme activity in different brain regions of rats, and it caused anxiogenic-like effects [22]. Lonare et al. [24] reported that exposure to imidacloprid inhibited AChE enzyme activity in the brain of rats, and that these changes in AChE activity, together with other neurotoxicity parameters, also decreased spontaneous locomotor activity and stimulated the pain sensation. In the present study, no behavioural tests were carried out to determine whether sulfoxaflor caused behavioural changes. The repeated exposures to sulfoxaflor at the concentrations evaluated in the current study did not cause any toxicity symptoms during the exposure periods. However, the toxicity symptoms such as loss of balance, erratic swimming and rapid gill movement were observed following the exposure to >3.51 mg/L (10% of 96 h LC_50_) sulfoxaflor without mortality. It can be concluded that sulfoxaflor may cause similar results on animal behaviour, considering previous studies that indicated a relationship between the changes in AChE enzyme activity and the behavioural alterations caused by neonicotinoids. Thus, it is obvious that evaluation of the neurotoxic potential of sulfoxaflor with its effect on behavioural changes in fish and nontarget animal species is important for risk assessment and environmental health.

Neonicotinoids have highly variable environmental half-lives ranging from minutes to several weeks in water [64]. Therefore, they have been determined in different kinds of water bodies, including surface water and groundwater [65]. Studies demonstrated that various neonicotinoids have been detected, ranging in concentration from 0.001 to 320 μg/L in aquatic environments [3,13]. No studies regarding the environmental concentration in water bodies of sulfoxaflor or its metabolites have been conducted until this time. Similar to neonicotinoids, sulfoxaflor has a variable half-life ranging from 37–88 days in aquatic systems under aerobic conditions and ranging from 103–382 days under anaerobic conditions [2]. It is obvious that interactions between sulfoxaflor and the environment have not been well revealed. Considering previous studies, sulfoxaflor might be found in aquatic ecosystems at similar concentrations to other neonicotinoids. Based on the present study’s findings, sulfoxaflor could produce a sublethal impact on zebrafish and other nontarget animal species via activation of AChE following exposure to the potential environmentally relevant concentrations.

## 5. Conclusions

The possible risk of environmental contamination due to the increasing use of neonicotinoid insecticides can create problems for human, animal and environmental health. Although there is clear evidence of the effects of other neonicotinoids on nontarget animal species in the literature, a limited number of studies are available related to the in vivo effects of sulfoximine containing neonicotinoids on other nontarget species. The present study demonstrated that sulfoxaflor acutely caused inductions of AChE enzyme activity in the brain and muscle tissues of zebrafish, in contrast to most of the other neonicotinoids. Thus, the inductions in AChE caused by sulfoxaflor might indicate that sulfoxaflor can be considered as a neuroactive compound for zebrafish. In addition, the tissue-specific response was not determined due to similar induction rates of AChE in both tissues. AChE induction might not be suggested as a biomarker due to the lack of a dose–response relationship for sulfoxaflor exposure. Many of the neonicotinoids cause behavioural changes and produce a neurotoxic response in fish and mammals through AChE inhibition. These findings highlighted the possible effects of sulfoxaflor on AChE enzymes in fish. Further studies are needed to clarify the actual toxic effects of sulfoxaflor on the nervous system by investigating main neurotoxicity parameters and behavioural alterations in nontarget animal species.

## Figures and Tables

**Table 1:** Lethal concentrations (LC_1–99_) of sulfoxaflor for zebrafish (*N* = 12, two replicates).

Lethal Concentrations	Sulfoxaflor (mg/L)	95% Confidence Limits	
		Lower	Upper
LC_1_	17.347	12.27	20.782
LC_5_	21.331	16.621	24.403
LC_10_	23.816	19.501	26.64
LC_15_	25.654	21.685	28.308
LC_50_	35.13	32.469	38.298
LC_85_	48.106	43.11	58.428
LC_90_	51.819	45.757	65.051
LC_95_	57.855	49.898	76.398
LC_99_	71.140	58.538	103.587
Slope ± SEM	7.592 ± 1.281		
Intercept ± SE	−11.734 ± 1.967		
χ^2^ value	3.873		
*p*	<0.05		

Control group (theoretical spontaneous response rate) = 0.000.

**Table 2:** Effects of sulfoxaflor on AChE-specific enzyme activity (U/mg protein) in the brain of zebrafish.

Exposure Periods	AChE Enzyme Activity/Brain			
	Group I (Control)	Group II (0.87 mg/L Sulfoxaflor)	Group III (1.75 mg/L Sulfoxaflor)	Group IV (3.51 mg/L Sulfoxaflor)
24 h	0.588 ± 0.015 bx	0.485 ± 0.034 cy	0.649 ± 0.041 abxy	0.737 ± 0.029 ay
48 h	0.569 ± 0.019 ax	0.548 ± 0.048 ay	0.571 ± 0.064 ay	0.490 ± 0.014 az
96 h	0.585 ± 0.021 cx	0.746 ± 0.019 bx	0.757 ± 0.020 bx	1.073 ± 0.062 ax

Values are expressed as means ± standard error. The letters a, b and c show differences among groups with different sulfoxaflor concentrations, and the letters x, y and z show the differences among groups at different treatment periods. Data shown with different letters are significantly different at the *p* < 0.05 level (*N* = 6).

**Table 3:** Effects of sulfoxaflor on AChE-specific enzyme activity (U/mg protein) in the muscle of zebrafish.

Exposure Periods	AChE Enzyme Activity/Muscle			
	Group I (Control)	Group II (0.87 mg/L Sulfoxaflor)	Group III (1.75 mg/L Sulfoxaflor)	Group IV (3.51 mg/L Sulfoxaflor)
24 h	0.699 ± 0.018 bx	0.650 ± 0.029 bx	0.685 ± 0.024 bz	0.832 ± 0.030 ay
48 h	0.662 ± 0.010 bx	0.674 ± 0.021 bx	0.804 ± 0.019 ay	0.823 ± 0.019 ay
96 h	0.671 ± 0.026 cx	1.03 ± 0.083 by	0.90 ± 0.028 bx	1.207 ± 0.041 ax

Values are expressed as means ±standard error. The letters a, b and c show the differences among groups with different sulfoxaflor concentrations, and the letters x, y and z show the differences among groups at different treatment periods. Data shown with different letters are significantly different at the *p* < 0.05 level *(N =* 6).

## Data Availability

The data presented in this study are available on request from the corresponding author.
